# Moving spiders do not boost visual search in spider fear

**DOI:** 10.1038/s41598-024-69468-3

**Published:** 2024-08-16

**Authors:** Miriam Becker, Nikolaus F. Troje, Filipp Schmidt, Anke Haberkamp

**Affiliations:** 1 Clinical Psychology and Psychotherapy, University of Marburg, Gutenbergstr. 18, 35032 Marburg, Germany; 2 York University, Toronto, Canada; 3 Justus Liebig University Giessen, Giessen, Germany; 4 Center for Mind, Brain and Behavior (CMBB), University of Marburg and Justus Liebig University Giessen, Giessen, Germany

**Keywords:** Human behaviour, Signs and symptoms

## Abstract

Previous research on attention to fear-relevant stimuli has largely focused on static pictures or drawings, and thus did not consider the potential effect of natural motion. Here, we aimed to investigate the effect of motion on attentional capture in spider-fearful and non-fearful participants by using point-light stimuli and naturalistic videos. Point-light stimuli consist of moving dots representing joints and thereby visualizing biological motion (e.g. of a walking human or cat) without needing a visible body. Spider-fearful (n = 30) and non-spider-fearful (n = 31) participants completed a visual search task with moving targets (point-light/naturalistic videos) and static distractors (images), static targets and moving distractors, or static targets and static distractors. Participants searched for a specified animal type (snakes, spiders, cats, or doves) as quickly as possible. We replicated previous findings with static stimuli: snakes were detected faster and increased distraction, while spiders just increased distraction. However, contrary to our hypotheses, spider targets did not speed up responses, neither in the group of control nor in the group of spider-fearful participants. Interestingly, stimuli-specific effects were toned down, abolished, or even changed direction when motion was introduced. Also, we demonstrated that point-light stimuli were of similar efficiency as naturalistic videos, indicating that for testing effects of motion in visual search, “pure” motion stimuli might be sufficient. As we do show a substantial modulation of visual search phenomena by biological motion, we advocate for future studies to use moving stimuli, equivalent to our dynamic environment, to increase ecological validity.

## Introduction

Motion attracts our attention. It is perceptually very salient^1^ as well as emotionally significant^2–4^. For example, when asked about the most scary and disgusting features of spiders, individuals with spider phobia most frequently report “movement of the spider”^5^. However, research on visual attention to fear-relevant stimuli has rarely considered motion, and even less so natural movement. Instead, experiments typically used static pictures or drawings^6–9^.

## Research on early attentional processes

Research on attentional processes in specific phobia has been vital to understand the etiology and maintenance of these disorders^10,11^. In case of acute threat, early attentional engagement enables the organism to protect itself (e.g. fight, flight). However, each startle response at the sight of a green stick that we mistook for a snake is wasting resources. Still, the gain of surviving an actual snake encounter seems to outweigh the costs of false alarms^12^. In anxiety disorders, this leads to hypervigilance—a constant monitoring of the environment for threat^13,14^.

Early attention processes for threatening and fear-relevant stimuli have been studied extensively using static images in visual search paradigms. In these paradigms, groups of stimuli are presented on the screen and participants have to indicate as fast as possible if and where a “target” stimulus is presented among the other (“distractor”) stimuli. By comparing response times for different constellations of target and distractor stimuli, attentional processes for different stimuli are compared. The experimental findings indicate two early attentional biases: Facilitated attentional capture and prolonged disengagement^15,16^. In these experiments, stimuli are labelled as fear-relevant (e.g. spiders in spider phobia), threatening (e.g. snakes), or neutral (e.g. mushrooms). The studies show that individuals with spider-phobia attend faster to fear-relevant stimuli (spiders) and take longer to disengage from them than individuals without spider-phobia^9,17–21^. Individuals with and without spider phobia attend faster to threatening stimuli (snakes) and need longer to disengage from them compared to neutral stimuli (mushrooms)^22–28^. However, these findings have not been reported in all studies, potentially because of methodological differences in task or measures of attention^9,16,19,29–33^.

### The role of movement

All of these findings for early attentional effects were obtained with static stimuli. This is potentially problematic, as many threatening and fear-relevant stimuli (e.g. animals) in our environment are typically encountered when moving. This also appears to impact the acquirement of specific fears: Infants spent more time looking at snake videos compared to videos of other animals but they did not make a difference between pictures of different animals^34^. Also, motion is well known to affect early attentional effects: For example, the onset of movement as well as biological motion capture attention in experimental settings^1,35,36^ and moving targets in chasing detection paradigms are faster identified among static distractors than vice versa^37–39^. Other findings point to a specific role of biological motion cues by showing that only upright, animate motion but no other motion triggers cued attention^40,41^. To isolate the effect of biological motion, the authors used point-light stimuli that consist of moving dots, each representing a joint^42^. Together, these dots visualize biological motion (e.g. of a walking human or cat) without a explicitly visible body^43–45^.To this day, the effect of biological motion on attention has been studied separately from the effects of emotional significance^41,46,47^, with no studies showing fear-relevant biological motion stimuli to individuals with specific phobia.

Specifically in spider phobia, the movement of spiders has been reported most frequently as anxiety-inducing factor^5^. However, only few experimental studies investigated the role of motion. For example, Vrijsen et al.^48^ modified the dot probe task – in which one of two simultaneously presented stimuli is followed by a target – by moving images across the screen instead of presenting a static image. However, this movement was no “biological” motion as the spiders did not move their legs and did not turn when their motion path described a curve. As in previous studies with static images, spider-fearful participants showed an attentional bias towards moving spider images compared to moving neutral stimuli. Schmidt et al.^3^ showed videos and images of different animals to participants with and without spider fear and obtained emotional judgements. Their results show that motion enhances the emotional evaluation (positive and negative) of animals, which is further pronounced for feared animals such as spiders; additionally, in line with qualitative reports^5^ it seems to be the unpredictability of spider motion that increases fear and disgust in individuals with spider-fear but not without spider-fear^49^.

### The current study

The present study was motivated by three research questions: First, can we replicate earlier findings of preferential processing of threatening/fear-relevant stimuli? Second, is there a specific effect of motion on attentional capture and disengagement? Third, does motion further boost the preferential processing of threatening/fear-relevant stimuli? In addition, we want to contrast the effects of “pure” biological motion in point-light stimuli with those of videos of moving animals.

Therefore, we presented threatening and fear-relevant stimuli in a visual search task to replicate earlier results on attentional capture and disengagement, and test the modulating effects of different types of motion. Participants had to identify a target among eight distractors by key presses. We chose to combine neutral and threatening/fear-relevant target/distractors, since this comparison made it possible to distinguish attentional capture from a difficulty in disengaging from threat.

### Hypotheses

1. *Influence of valence on target search*. (1a) *Target valence*: Threatening targets should be found faster compared to neutral targets in the group of non-fearful participants (RTs: cat/dove target > spider/snake target), and fear-relevant targets should be found fastest in the group of spider-fearful participants (RTs: cat/dove > snake > spider). (1b) *Distractor valence*: We expected the reversed effect of distractor valence on target search, with threatening and/or fear-relevant stimuli slowing the search compared to neutral stimuli (non-fearful group: spider/snake > cat/dove; spider-fearful group: spider > snake > cat/dove).
2. *Motion*: The presence of motion was expected to affect target identification (i.e., faster search for moving targets, slower search when distractors move).
3. We hypothesized that motion and stimulus valence would interact, such that moving targets would increase the attentional bias towards threatening/fear-relevant targets (i.e., the search advantage of threatening targets increases when targets move). We expected this difference for fear-relevant targets to be more pronounced in the spider-fearful group.
4. We expected slower disengagement from threatening/fear-relevant distractors which should be even slower for moving distractors. For fear-relevant distractors this difference should be more pronounced in individuals with spider-fear.
5. We aimed to isolate the effect of biological motion on early attentional processes by testing point-light stimuli, allowing us to compare our findings for “pure” biological motion with those for videos of moving animals.

## Methods

### Participants and Power Analysis

A total of 61 participants were included in the study (30 spider-fearful, 31 non-fearful individuals). The sample size was determined by a power simulation using R. The power analysis incorporated the factors group (spider-fearful vs. non-fearful) × target/distractors type (snake/neutral, spider/neutral, neutral/neutral, neutral/snake, neutral/spider) × motion (targets moves, distractors move, all static) × motion type (natural vs. point-light). For target/distractors type differences in static images we expected the effect sizes to be medium to large^50^ based on existing literature^7,9,19^. Since there is no comparable research using videos or point-light stimuli, we estimate the effect sizes to be small to medium^51^. To obtain a 1 − β of 0.80 at an α = 0.05 for the effects of group × target/distractors type and the main effect of motion n = 10 per group would have been sufficient. To detect the three-way interaction with the same 1 − β of 0.80, we would have needed to recruit at least 80 subjects per group. Given that recruiting spider-fearful subjects for a lab study, which forces them to encounter the object of their fear without getting some form of treatment in return, is very hard, we set out to recruit n = 30 subjects per group instead. Thus, the resulting sample size of n = 30 per group lead to small three-way interactions being underpowered in this study (1 − ß of 0.40).

Participants were recruited via a mailing list of the local university and leaflets distributed on campus. They received 15 € or course credit for payment. Participation requirements were normal or corrected-to-normal vision, and no known psychotic disorder diagnosis (current or prior) or previous treatment for spider phobia. To assess eligibility, participants completed the following questionnaires via the online tool Unipark^52^: Spider Phobia Questionnaire (SPQ^53,54^), Fear of spiders questionnaire (FAS^55,56^), Snake Anxiety Questionnaire (SNAQ^53,54^), and the Beck Depression Inventory (BDI-II^57,58^). Additionally, we used the specific phobia section of the Diagnostic Interview for Psychological disorders (DIPS^59^) to test for the clinical relevance of spider phobia.

185 individuals participated in the study. Participants with BDI sum scores > 13 (indicating “mild depression”) were excluded (18 participants). Only participants with SNAQ scores ≤ 50^th^ percentile were included (131 participants) to obtain a sample with low to average snake fear. To obtain two substantially different experimental groups, participants were separated into spider-fearful (≥ 75th percentile) and non-spider-fearful (≤ 25^th^ percentile) according to their SPQ values (excluding 18 participants). In total, 113 participants were eligible. 46 individuals did not finish their participation by discontinuing the questionnaire (5) or missing their lab appointment (41). After data collection, we excluded six of 67 participants: three participants because of high error rates (> 33% per block), two participants for insufficient language proficiency (i.e., understanding of questionnaires and task instructions was uncertain), and one because of inconsistent diagnostic results (low scores of spider fear in the questionnaires, but high spider fear reported in the interview). The final sample consisted of 30 spider-fearful (age: *M* = 25.23, *SD* = 4.45, range 19–37; gender: 25 female, 4 male, 1 diverse) and 31 non-fearful individuals (age: *M* = 24, *SD* = 5.14, range 19–43; gender: 22 female, 9 male).

### Materials and apparatus

#### Questionnaires and Interviews

*Beck Depression Inventory-II*. The BDI-II is a 21-item scale measuring depression symptom severity^57,58^. Per symptom participants choose one of four statements, which are assigned values ranging from 0 to 3. The German version is suggested to be reliable and valid^57^. Internal consistency is excellent for individuals with depression (α = 0.93) and individuals without mental disorders (α = 0.90).

*Spider Phobia Questionnaire***.** The SPQ is a 31-item scale measuring spider phobia symptom severity via a dichotomous response format (“Yes” vs. “No”)^53,54^. It is widely used to measure spider phobia severity and has been shown to be reliable and valid^53^. The internal consistency is satisfactory (α = 0.83).

*Fear of Spiders Questionnaire*. The FSQ is an 18-item scale measuring spider phobia symptom severity^55,56^. Items are rated on a 7-point Likert scale ranging from 0 = “not at all” to 6 = “very much”. It is frequently used to corroborate findings from the SPQ ^60^ and has shown good psychometric qualities ^55^. The internal consistency is excellent (α = 0.96).

*Snake Anxiety Questionnaire*. The SNAQ is a 30-item scale measuring snake phobia symptom severity via a dichotomous response format (“Yes “ vs. “No “)^53,54^. It has been shown to be reliable and valid^53^. The internal consistency is satisfactory (α = 0.83).

*Diagnostic Interview for DSM-V*. The DIPS is a structured interview for the diagnosis of mental disorder^59^. We used the section on specific phobias. The reliability for the specific phobia section (κ = 0.68) and validity are considered good^61,62^.

#### Stimuli

Stimuli were natural and point-light videos, as well as pictures of spiders, snakes, doves, and cats. We used doves and cats in contrast to previous studies which used plants or arthropods (e.g. flowers/butterflies) since the latter do not move on the ground like spiders.

The natural video stimuli consisted of eight different animals per category (i.e. eight different cats). The videos were selected from stock footage databases. In all videos, the animal moved straight ahead at the center of the screen with no or little camera motion. The average luminance of the videos was equalized using the SHINE_color toolbox for Matlab^63,64^.

Point-light videos consist of moving dots, each representing a joint^42^, that simulate biological motion, for example, a walking human or cat^43,65^. For point-light stimuli, we used four videos per animal category with different walking directions (left to right, right to left, upper right to lower left corner, lower left to upper right corner). Point-light stimuli of a walking human and dove were used from previous studies^44,65^. Snake videos were provided by Vanessa LoBue^66^. The spider videos were created in Blender^67^ based on a commercially purchased 3D spider model.

**Figure 1:**
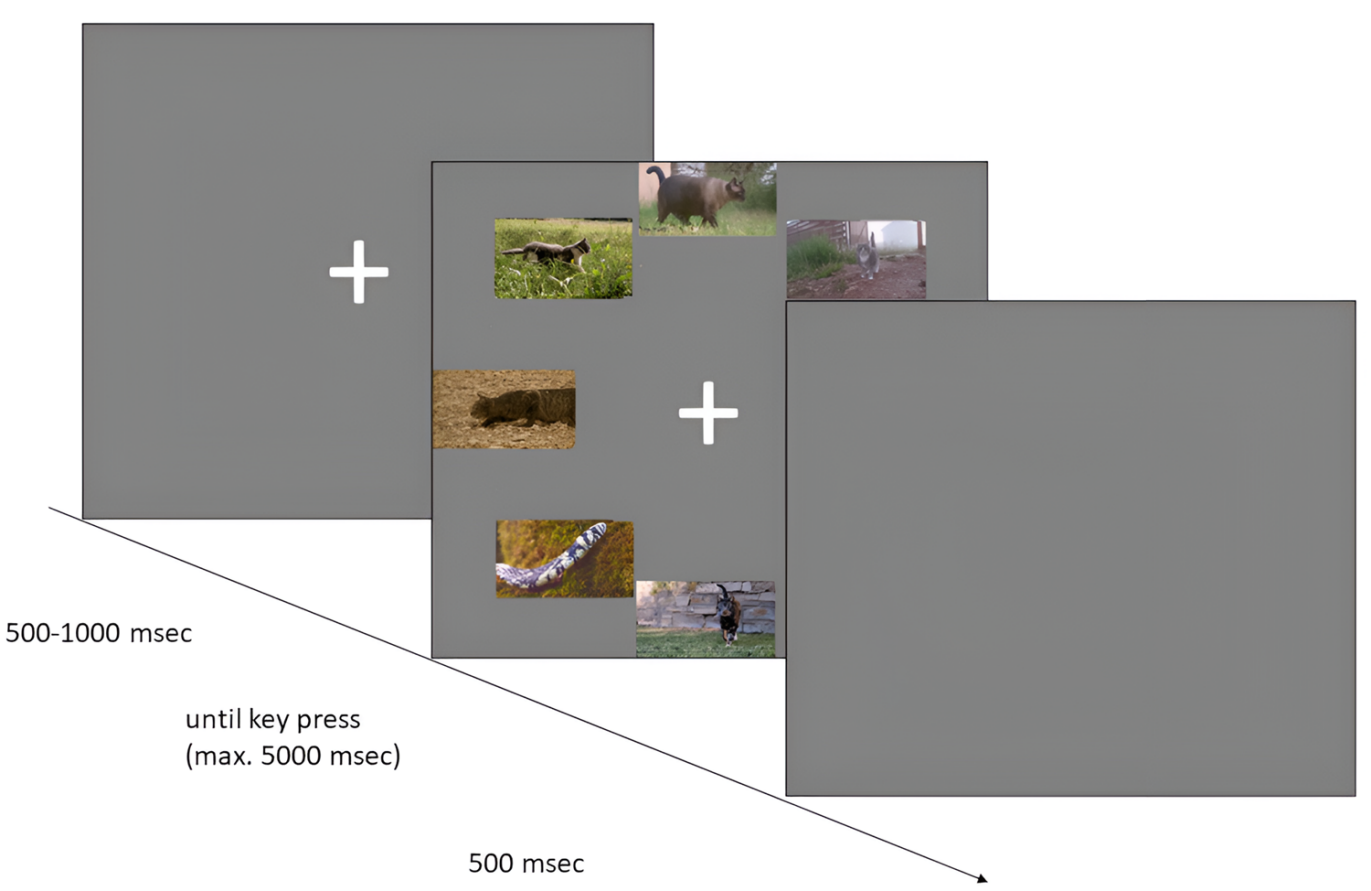
Example Trial. The example trial shows a snake among cat distractors in the natural stimuli block.

Stimuli were presented with Psychopy Builder, Version 2021.1.2^68^ on a 60 × 33 cm screen with a 1920 × 1200 pixel resolution. All stimuli measured 195 × 109.5 pixel and the distance between participant and screen was approx. 50 cm. In each trial, eight stimuli were presented in a circular arrangement (300 pixel radius) with equal distance between stimuli and a distance of 9.67 degree of visual angle between stimuli centers and screen center. Stimuli were presented on a light grey background (cf. Fig. 1).

### Design

The experiment consisted of 18 practice trials and two experimental blocks with 180 trials each (2 × 120 target-present + 2 × 60 target-absent trials = 360 trials total). One block presented natural videos and pictures, the other block point-light-stimuli. The order of both blocks was randomized. Each block featured 15 target-present trial conditions (5 × 3 × 2 design): target/distractors type (either spider/neutral, snake/neutral, neutral/spider, neutral/snake, or neutral/neutral), motion (target moving, distractors moving, or all static), and motion type (natural or point-light stimuli). Each condition was repeated eight times with pseudorandomized order of videos/pictures. One-third of all trials (120 out of 360) were target-absent to ensure that participants did not simply press the answer key without looking for the target, similar to previous visual search experiments^7,9,30,69^.

### Procedure

The study was approved by the Ethics Committee of the Department of Psychology of the University of Marburg (2020-53 k). All participants provided informed consent and were treated in accordance with the ethical guidelines of the German Psychological Society. The study was preregistered at the Open Science Framework (https://osf.io/4bseu/). After completing the questionnaires via the online tool Unipark, eligible participants came to the lab for testing. First, they completed the visual (category) search task: Participants were told to search for a particular type of animal per block (e.g. “Search for the spider!”) and to press the right arrow key as soon as they found the animal and the left arrow key on target absent trials. A fixation cross (duration 500–1000 ms) at the center of the screen preceded each trial. Subsequent stimulus presentation ended after participants’ response or 5000 ms without response, and a blank screen appeared for 500 ms (Fig. 1). We chose the category search task for two reasons. First, it allowed us to include target-absent trials, which is not possible in a traditional odd-one out task^9^; second, we wanted to prevent participants’ responses to be dominated by motion differences between target and distractors.

The visual search task was followed by a rating of all videos (n = 46) and pictures (n = 46) on 7-point Likert-Scales for valence, arousal, disgust, and anxiety. Finally, we assessed clinical criteria for spider phobia using the semi-structured interview DIPS.

### Statistics

For statistical analysis, raw data for reaction times and error rates (wrong or no answer) were averaged across repeated measures into mean values per participant and category. Only target-present trials were included in the analysis. We used R version 4.1.1 to perform a four-way mixed ANOVA with group (2: spider-fearful, non-spider-fearful) as in-between-factor and within-factors of target/distractors type (5: spider/neutral, snake/neutral, neutral/spider, neutral/snake, neutral/neutral), motion (3: target moving, distractors moving, all static), and motion type (2: natural, point-light stimuli). To answer the more specific hypotheses of H3a and b, we calculated difference scores between target/distractors types and neutral target/neutral distractor types, and compared them between motion conditions, resulting in the following within-factors: target/distractors type difference (4) and motion difference (2). Error rates per block were inspected using a three-way mixed ANOVA with group (2) as in between factor and within-factors animal (4) and motion type (2). For each rating variable (anxiety, arousal, disgust, valence), a four-way mixed ANOVA with group (2) as in between factor and within-factors animal (4), motion (2) and motion type (2) was calculated to verify the manipulation. For tests violating the sphericity assumption, we used the Greenhouse–Geisser correction, and the Bonferroni-Holm-Method to correct for alpha-error accumulation in post-hoc tests.

## Results

### Questionnaires

Corresponding to our eligibility criteria, spider-fearful individuals scored significantly higher than non-fearful individuals on the spider questionnaires (FAS and SPQ). However, they did not differ in their depression (BDI) or snake fear (SNAQ) scores (Table 1). Regarding the DIPS, 17 out of 30 spider-fearful participants met all 7 criteria for spider phobia; 8 met 6 criteria, 3 met 5 criteria and 2 met 1 criterion; and non-fearful participants did not meet any criteria for specific spider phobia.

**Table 1:** Mean questionnaire scores per group.

Questionnaires	Non-Spider-fearful		Spider-fearful		*p*
	*M*	*SD*	*M*	*SD*	
BDI	3.00	3.45	3.87	3.95	.365
SNAQ	3.42	2.41	3.70	2.38	.649
FAS	25.94	18.76	90.73	18.96	< .001
SPQ	2.97	2.39	21.70	3.80	< .001

*p* = *p*-value of one-way ANOVA. M = mean, SD = standard deviation. BDI = Beck Depression Inventory. SNAQ = Snake Anxiety Questionnaire. FAS = Fear of Spider Questionnaire. SPQ = Spider Phobia Questionnaire.

### Error rates

For all target present trials, errors ranged, comparable with previous studies on visual search, between 0.4 and 15% (average 3.7%)^9,19,55^. The groups of spider-fearful and non-spider-fearful participants did not differ in their error rates (*p* = 0.741). Interestingly, blocks with point-light stimuli had fewer errors compared to blocks with natural stimuli (*p* < 0.001, see Supplementary A). This suggests that targets can be better identified with less visual “clutter” like background and texture even though they are reduced to point lights. Error rates per animal depended on motion type (*p* < 0.001): Among natural stimuli, snake target blocks yielded fewest errors (*p* ≤ 0.03). Also, among point-light stimuli, snake target blocks yielded fewer errors than dove and spider target blocks (*p* ≤ 0.026). Thus, snakes seem to have a perceptual advantage in both motion types, which is in line with prior research^70–73^.

### Stimulus Rating (Manipulation Check)

Motion as well as motion type affected stimulus ratings. Video stimuli were rated as more arousal- (*p* < 0.001), and disgust-inducing *(p* = 0.01), and as more positive (*p* = 0.049) compared to static stimuli. Anxiety ratings for videos compared to images failed to reach significance (*p* = 0.08). Point-light stimuli elicited less anxiety (*p* < 0.001), arousal (*p* < 0.001) and disgust (*p* < 0.001), and were rated as more positive (*p* = 0.004). As expected, spider-fearful participants rated spiders as most arousal-, anxiety-, disgust-inducing, and most negative (post-hoc tests, *p* ≤ 0.05) compared to other animals, and compared to individuals without spider fear (*p* < 0.001; for details see Supplementary B). However, non-spider-fearful participants rated spiders as more negative than snakes on all four rating variables (*p* ≤ 0.05). To test a potential effect of the unexpected differences between snake and spider ratings among non-fearful individuals, we conducted exploratory correlation analyses between the (spider/snake) stimulus ratings and reaction times to (spider/snake) targets – however, with no significant results (for details see Supplementary C). Importantly, motion and motion type did not affect group-differences for any rating (*p* > 0.1). And contrary to our expectations, disgust and valence ratings for the natural neutral animals (cats/doves) were not identical (*p* ≤ 0.05). Besides, ratings were in accordance with our expectations. Thus, reaction time differences cannot be attributed to insufficient manipulation of anxiety, disgust, arousal, and valence.

### Category search task

#### (1a) Snake but not spider targets speed up search irrespective of spider-fearfulness

**Figure 2:**
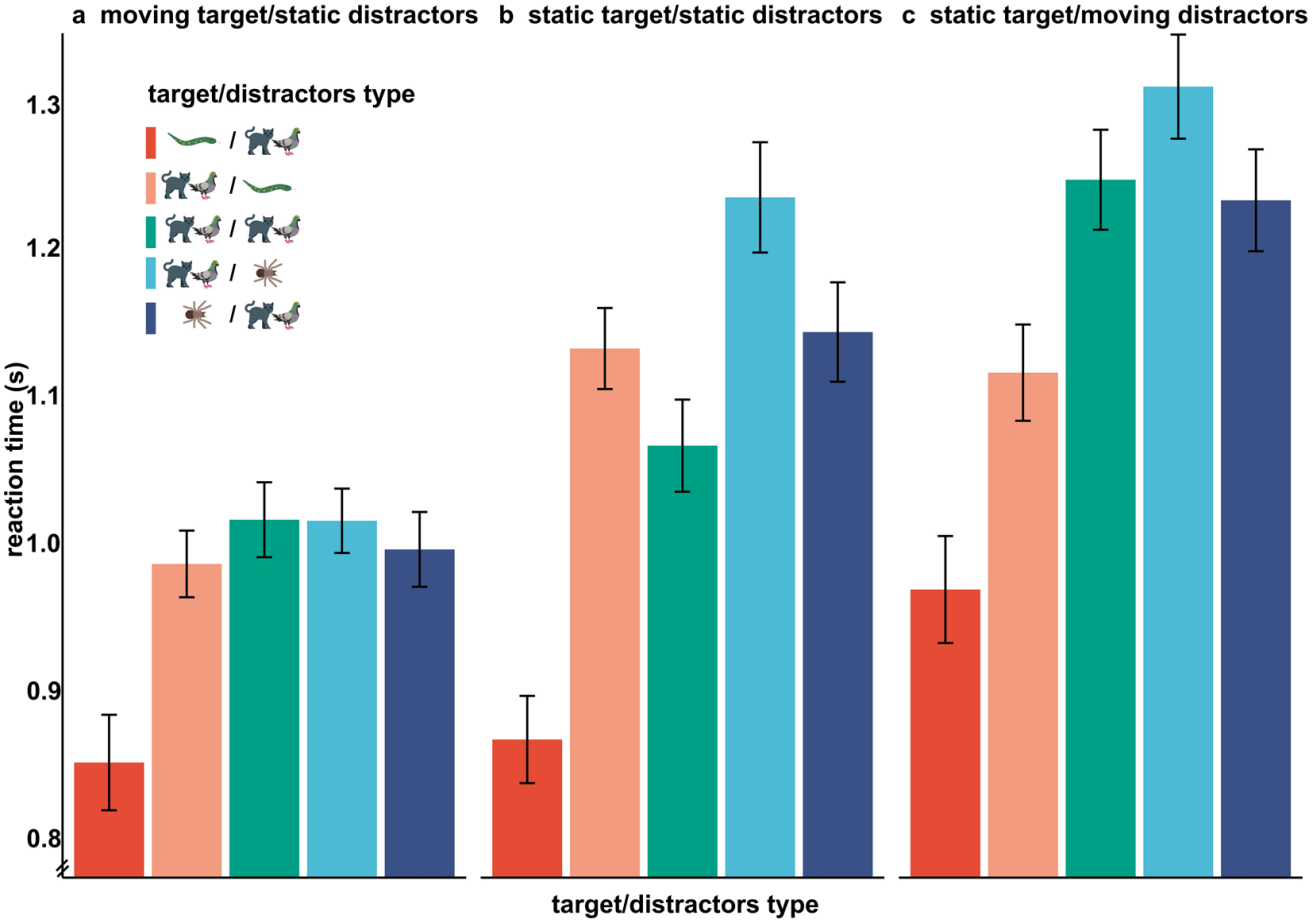
Effects of target/distractors type within motion conditions. *Note*. Mean ± standard error of mean (SEM).

To compare our results to previous research, we first focus on target/distractors type differences for the motion condition “static target/static distractors” across groups (see Fig. 2b). In contrast to our expectations, spider targets slowed down search (*t*_(121)_ = −3.32, *p* = 0.002; dark blue bar in Fig. 2b), while, interestingly, snake targets elicited faster reaction times than neutral targets (*t*_(121)_ = −10.7, *p* < 0.001; dark red vs. green bar in Fig. 2b). The group × target/distractors type interaction barely failed to reach significance (*F*_(3.16, 186.22)_ = 2.352, *p* = 0.056), but no main effect of group was found within any of the different target/distractors types (*p* > 0.1, see Supplementary C). This implies that spider-fearfulness did not modulate the reactions to different target/distractor combinations.

#### (1b) Spider and snake distractors slow down irrespective of spider-fearfulness

As expected, snake and spider distractors slowed down search compared to neutral distractors (*t*_(121)_ = 3.35, *p* = 0.002, *t*_(121)_ = −8.07, *p* < 0.001; light blue/light red bars vs. green bar in Fig. 2b). However, spider-fearfulness did not affect reaction times to target/distractor combinations.

#### (2) Motion: targets speed up search, distractors slow down search

**Figure 3:**
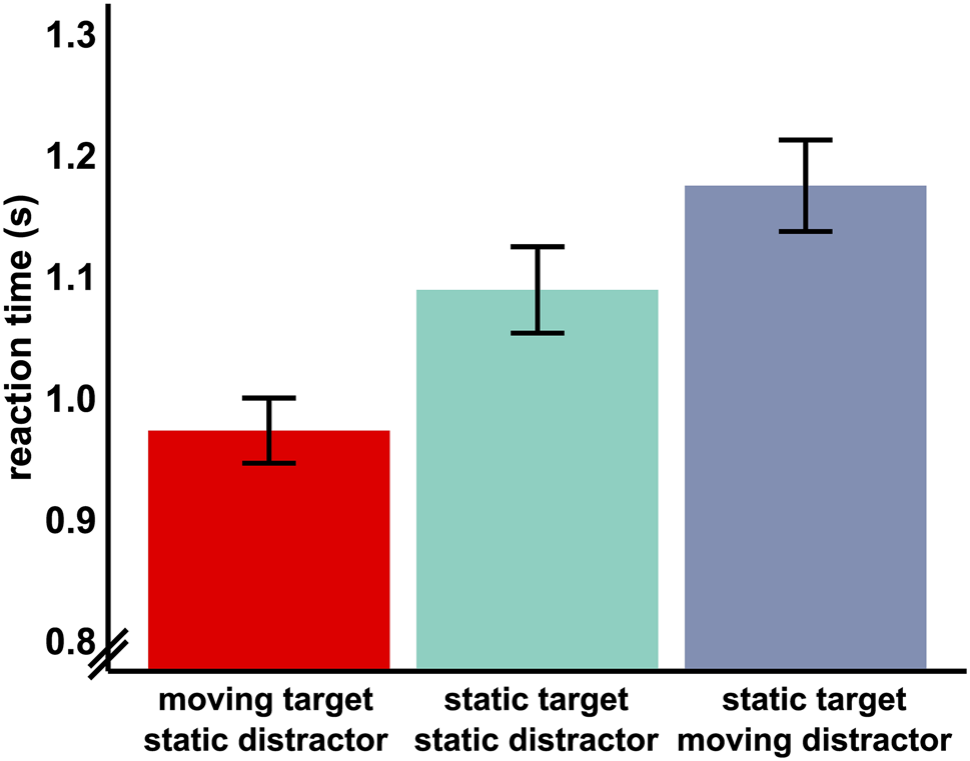
Effect of motion. *Note*. Mean ± standard error of mean (SEM).

Motion significantly affected reaction times (*F*_(1.78, 105.30)_ = 188.35, *p* < 0.001). Post-hoc tests showed that moving targets sped up target search (*t*_(609)_ = 14.3,* p* < 0.001) by about 117 ms whereas moving distractors slowed down target search (*t*_(609)_ = 10, *p* < 0.001) by about 80 ms, as compared to static stimuli (Fig. 3).

#### (3a + 3b) Motion influences but does not amplify effects of target/distractors type

We detected no effect of motion on the target/distractors type × group interaction (*F*_(8, 472)_ = 0.83, *p* = 0.579). Due to our sample size, the detection of this three-way interaction was underpowered (see limitations for further discussion). The interaction of motion × target/distractors type was significant (*F*_(8, 472)_ = 21.53, *p* < 0.001). Specifically, the influence of motion on reaction times was observed for all different target/distractors types (*p* ≤ 0.005), with two exceptions. First, if the target was a snake, its motion did not speed up search compared to when it was static (*t*_(121)_ = 1.27, *p* = 0.223; dark red bars in Fig. 2a vs. b). Second, if the distractors were snakes, their motion did not slow down search compared to when they were static (*t*_(243)_ = −0.898,* p* = 0.371; light red bars in Fig. 2c vs. 2b). But, as expected, reaction time to target/distractors types without snakes was slowed down by moving distractors and speed up by moving target (green and blue bars in Fig. 2).

*Target motion abolishes target/distractor effects except for snake targets*. If targets were in motion, all differences between target/distractors types disappeared except the faster detection of snake targets (*t*_(121)_ = −9.91, *p* < 0.001; Fig. 2a). Specifically, neither snake nor spider distractors slowed down search compared to the neutral target/distractors types when targets were moving (spider distractors: *t*_(121)_ = 0.05, *p* = 0.957). Rather snake distractors sped up search compared to neutral target/distractors types (*t*_(121)_ = −2.22, *p* = 0.035; light red vs. green bar in Fig. 2a).

Contrary to our hypotheses, motion of spider targets did not amplify the effect of the static condition (*t*_(121)_ = 1.36, *p* = 0.204). Rather moving spider targets were found as fast as moving neutral targets (dark blue and green bars in Fig. 2a). In contrast to our hypotheses, introducing motion of the snake target did not further speed up responses, either. Rather, the speeded detection of snakes for moving target/static distractors type was smaller, which failed to reach significance barely (RT_neutral-snake target_: *M*_static_ = 0.199, *SD*_static_ = 0.13, *M*_move_ = 0.164, *SD*_move_ = 0.111, *p* = 0.062; dark red bars in Fig. 2b vs. a).

*Distractor motion does not enhance distraction by spiders*. In line with our hypotheses, moving spider distractors did slow down search more than neutral target/distractors types (*t*_(121)_ = −3.19, *p* = 0.003; light blue vs. green bar in Fig. 2c). However, the distraction by moving spiders was smaller compared to static spider distractors when compared to the neutral target/distractor types in the same condition (RT_neutral-spider distractor_: *M*_static_ = −0.168, *SD*_static_ = 0.176; *M*_move_ = -0.063, *SD*_move_ = 0.159, *p* < 0.001; difference between light blue and green bar in Fig. 2b vs. c). Also, the response to targets among moving snake distractors was faster (*t*_(121)_ = −6.87, *p* < 0.001) than to neutral target/distractors types (light red vs. green bar in Fig. 2c). This is possibly due to a relatively large increase in reaction time for neutral target/distractor types from static to moving distractor condition. If distractors moved, spider targets did not affect search (*t*_(121)_ = 0.63, *p* = 0.568; dark blue vs. green bar in Fig. 2c).

#### (4) Point-light stimuli speed up search compared to natural stimuli

**Figure 4:**
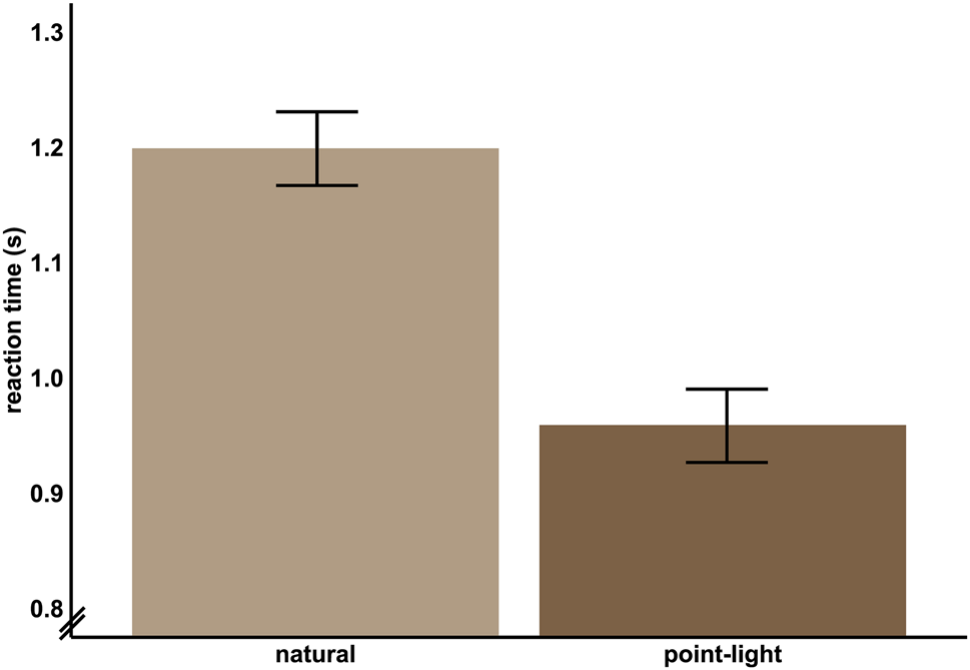
Effect of motion type. *Note*. Mean ± standard error of mean (SEM).

There was a significant main effect of motion type (*F*_(1, 59)_ = 228.05, *p* < 0.001). In fact, point-light stimuli yielded faster reaction times than natural videos (Fig. 4). For further exploratory analysis see Supplementary C.

## Discussion

### Summary of findings

To allow for a comparison with previous research our experiment also featured static stimuli. In line with our hypothesis for static stimuli, snake targets sped search up, while snake and spider distractors slowed search down. However, contrary to our hypotheses, spider-fearfulness had no effect on reaction times and spider targets did not speed up search but slowed it down.

In addition to frequently used pictures of animals, we employed naturalistic as well as point-light video stimuli. As expected, moving targets were identified faster than static targets, and moving targets prolonged search times compared to static distractors. Additionally, point-light stimuli were identified faster than natural stimuli.

Also, attentional capture by snake targets and distraction by spider distractors persisted when stimuli were moving. But unexpectedly, motion did not enhance these effects: Moving snakes sped up search to the same extent as static snakes. Moving spiders did even slow down search less than static spiders. Finally, contrary to our hypotheses, moving spider targets did not speed search up more than neutral targets, and moving snake distractors did not slow search down more than neutral distractors—rather they sped it up.

#### Increased distraction by spiders

Distraction by spiders in our experiment is in line with previous research on prolonged disengagement by threatening stimuli^7,26^. Surprisingly, the effect was not stronger for spider-fearful participants, which is in contrast to prior research demonstrating prolonged disengagement from phobic stimuli^9,19^. Also, we did not replicate earlier findings of a faster search for static spider targets by spider-fearful individuals^9,17,18,21^, adding to previous studies that failed to observe this effect^6,19,22,69,74^. Instead our participants reacted slower to pictures of spiders as targets^19^.

Since our stimuli did induce significantly different levels of anxiety, disgust, arousal, and valence in spider- and non-spider-fearful participants, the absence of a group difference is not explained by a lack in induction of emotion evaluation. However, our failure to replicate previous findings might be explained to some extent by differences in task: The majority of previous studies did use different task instructions where participants search for a picture, which is different from the others (e.g. spider among butterflies), instead of being instructed to search for a specific type of stimulus (e.g. spider)^9,17,18^. As the convergent validity between visual search tasks is rather poor^75^, minor task differences might explain the absence of attentional capture by spider targets in spider-fearful participants in our study. Importantly, there are no reliability or validity coefficients for studies using spiders or other specific phobia stimuli. Even if task differences modulate the effect size, our study supports previous accounts that attentional capture by spiders is not a stable effect and therefore cannot be replicated across all visual search tasks^9^.

Another possible explanation for the absence of a faster target identification of spiders comes from the Snake Detection Theory^76^. Even though our visual system was evolutionary optimized to detect both snakes and spiders, we and other mammals adapted primarily in response to selection pressure from the more dangerous snakes. Indeed, a number of previous experimental studies showed that snakes are detected rapidly, while interference by spiders is only relevant at later stages of the attentional process, e.g. during disengagement^77–79^. Considering that our task aimed at early visual attention, our findings might be interpreted as evidence for the Snake Detection Theory.

#### Faster search for snakes and distraction by snakes

Snakes were identified faster than neutral targets and static snake distractors slowed search down, replicating earlier findings with static snake stimuli^23,26,27^. How can we explain that the snake-in-the-grass effect is more stable than the attentional interference effect, induced by spiders? In line with others, we suggest that a major factor is visual complexity: Spiders are visually more complex than snakes, so that the snake targets have a perceptual advantage^70–73,80^ and pop out more than spiders. Also, snake distractors are more similar to each other, and might therefore provide a more homogeneous background for visual search which is easier to ignore compared to the more heterogeneous spider distractors. These explanations in terms of perceptual rather than emotional differences are supported by the emotional judgements, which show that snake stimuli did not induce more anxiety, arousal, disgust or negative valence than spiders. These visual advantages are—again—in line with the Snake Detection Theory^76^. If snakes did indeed exert a higher selection pressure on the mammalian visual system than spiders, we might expect better discrimination for them.

#### Point-light and natural video stimuli show similar response times

Our findings show that point-light stimuli are sufficient to study early attentional processes: They were as effective as natural videos in inducing a motion effect as well as demonstrating a distraction by spiders and an attentional capture by snakes. We suggest that this shows once again the strong saliency of motion^35,36,81^ and effectiveness of point-light stimuli^41,43–45^, which compensates for the absence of features such as color and texture. Notably, the emotional valence of point-light stimuli was lower compared to video stimuli, so that point-light stimuli might be less effective in studies mainly focusing on emotional (top-down) effects on attention. Therefore, in clinical research, point-light stimuli would probably underestimate the effect of emotional valence. Still, different point-light animal stimuli induced different emotional ratings, so that for experimental research, point-light stimuli allow to study the influence of biological motion and its threat evaluation on attentional processes across different animals without confounding visual features (color, texture).

#### Motion affects reaction times

In general, motion had the expected effects on overall reaction times, with moving targets that were found faster and moving distractors that slowed search down. This supports existing evidence for a strong effect of motion on attentional processes^36,37^. Interestingly, in our study the motion effect was not strong enough to boost the already faster identification of snake targets or the slower identification of targets among snake distractors. Furthermore, introducing motion for targets eliminated all differences between different target/distractors types, with the exception of snake targets. Thus, only the snake-in-the-grass effect is strong enough to overcome the effect of motion. Again, both findings might be explained in terms of accounts that preferential processing of snakes is not (only) due to their evaluation as threatening or due to their motion, but rather due to their visual form^72^.

Contrary to our expectations, motion did not increase the target/distractors type differences^3^. In fact, motion rather reduced (i.e., the increased distraction by spider distractors and the speeded detection of snake targets), abolished (i.e., slower reaction to spider compared to neutral targets) or changed direction of target/distractors type effects (i.e., static snake distractors distract, moving snake distractors speed up search). Our findings suggest that previously reported effects of static threatening stimuli might not translate to (biological) motion stimuli.

As many threatening and fear-relevant stimuli in our environment are typically encountered when moving, specifically animals, static stimuli are of limited ecological validity. Therefore, we advocate for future studies to use moving stimuli, equivalent to our dynamic real-world environment, to obtain experimental findings that are relevant for and can be translated to everyday life.

### Limitations and strengths

To distinguish between effects of motion and valence, we used mixed motion conditions (a moving target between static distractors and vice versa). Including an all-motion condition (moving target/moving distractors) would have allowed to disentangle the effects of motion in general vs. the effects of a difference in biological motion between target and distractors. A further limitation of our study is the absence of measurements that would allow us to map the time course of attention. For example, future studies might track eye movements to study the reaction time differences in more detail.

A strength of our study is the natural stimulus material: To achieve higher ecological validity, we chose videos and images of animals within their natural habitat. Although we did not include any videos with (much) camera motion or occluded animals, which might have been even more realistic, our videos featured different background textures. Future studies might take our approach one step further by investigating visual search for different animals within complex, naturalistic scenes (i.e., using the same background for all stimuli instead of search arrays of separate images/videos). Possible stimuli could include naturalistic scenes, as employed in free-viewing scenarios^3^. Another possible step would be using virtual reality^82,83^ or real-life search tasks^84^, which enables the participant to move within the scene. All of these approaches can help bridging the gap between lab research and everyday life.

To the best of our knowledge, our study is the first use threat-relevant biological motion stimuli in a visual search paradigm. This opens the opportunity to study the effects of “pure” biological motion without potential confounds like background texture or color of the animal.

Classic visual search tasks have been criticized over the years for various reasons that warrant a short discussion here. For example, reaction time differences in visual search task sometimes seem to depend more on arousal differences than on negative affect^85^. In the case of our study however, fear-relevant and threatening stimuli both induced more arousal and negative affect (valence, disgust, anxiety) than neutral stimuli. Still, future studies might use eye-tracking to study the time course of attention allocation in more detail.

We included target-absent trials (1/3 of all trials) to minimize the occurrence of false positive responses. This might have led to a more pronounced habituation towards the presented stimuli or to a response bias as measured by reaction times^86,87^. However, since each stimulus category was featured equally often in target-absent trials, any response bias or habituation should be the same across all stimulus categories over the complete timescale of the experiment. Nevertheless, over short timescales this could have potentially led to different levels of stimulus habituation.

Given the medium sample size (n = 61) of our study, some of our results should be interpreted with caution. 1 − ß for main effects and two-way interactions was sufficient (≥ 0.80). However, in our power analysis, we estimated the effects of motion to be small to medium, so that our design was not suited to reliably detect small interaction effects (e.g. group × target/distractors types × motion). Specifically, the absence of a group × target/distractor types × motion interaction should be interpreted with caution. Since our power to detect this interaction was limited (1 − ß = 0.40), we cannot draw a definite conclusion about the influence of motion on group × target/distractors types effects. Therefore, we cannot answer the question, whether moving animals elicit different attentional capture processes depending on spider-fearfulness when compared to static pictures. Because of this limitation, future replication studies should aim for larger sample sizes if possible, for example, by relying on online data collection.

### Conclusion

Our study investigated the influence of biological motion on attentional capture by and the prolonged disengagement from threatening stimuli. Our results demonstrate a stable snake-in-the-grass effect for moving and static stimuli. However, contrary to our hypotheses, spider-fearfulness did not modulate attentional processes—and distraction by spiders was even less pronounced when using moving compared to static stimuli. Also, we found no significant difference between the visual search for moving spiders compared to neutral targets. Our results demonstrate that results with static stimuli might not translate easily to moving stimuli. Future research should investigate whether the snake-in-the-grass effect is indeed the only attentional capture phenomenon that is preserved with moving stimuli. To test this, the effects of basic visual features (shape/motion) must be dissociated from the effects of biological motion and emotional valuation. Despite our rather nuanced results, our findings open a new avenue of research by using visual search to investigate the role of biological motion in the evaluation of threat, which will help to increase the translation from the lab into everyday life.

### Supplementary Information

Supplementary Information.

## Data Availability

The datasets and code used for statistical analysis, and the experimental code are available at the OSF (https://osf.io/8jy75/). Stimuli used in the study are available from the author(s) on reasonable request.
