# Supplementary material for: Moving spiders do not boost visual search in spider fear
Source: Sci Rep. 2024 Aug 16;14:19006. doi: 10.1038/s41598-024-69468-3 (PMC11329515; doi:10.1038/s41598-024-69468-3)
Supplement: Supplementary file 1 — Supplementary Information. [file 41598_2024_69468_MOESM1_ESM.docx]

**Supplementary for the manuscript “Moving spiders do not boost visual search in spider fear”**

Miriam Becker^1^, Nikolaus F. Troje^2^, Filipp Schmidt^3,4^ & Anke Haberkamp^1,3^

^1^University of Marburg, Germany

^2^York University, Toronto, Canada

^3^Justus Liebig University Giessen, Germany

^4^Center for Mind, Brain and Behavior (CMBB), University of Marburg and Justus Liebig University Giessen, Germany

**A: Error rates per block**

**Table S1**

*Error rates per block*

|  | natural | | point-light | |
| --- | --- | --- | --- | --- |
| targets | *M* | *SD* | *M* | *SD* |
| cat | 2.01 | 2.35 | 0.62 | 1.02 |
| dove | 1.89 | 1.70 | 1.02 | 1.34 |
| snake | 0.80 | 1.01 | 0.38 | 0.55 |
| spider | 1.33 | 1.45 | 0.74 | 0.98 |

*Note.* *M* = mean, *SD* = standard deviation.

**B: Means of Ratings and Reaction Time**

**Table S2**

*Ratings per animal*

|  | anxiety | | arousal | | disgust | | valence | |
| --- | --- | --- | --- | --- | --- | --- | --- | --- |
| animals | *M* | *SD* | *M* | *SD* | *M* | *SD* | *M* | *SD* |
| cat | 1.20 | 0.46 | 1.50 | 0.99 | 1.15 | 0.30 | +1.27 | 0.80 |
| dove | 1.11 | 0.29 | 1.34 | 0.80 | 1.45 | 0.58 | +0.71 | 1.10 |
| snake | 1.83 | 0.92 | 1.91 | 1.01 | 1.84 | 1.02 | +0.21 | 1.19 |
| spider | 3.40 | 1.8 | 3.53 | 1.89 | 4.14 | 1.83 | -1.73 | 1.48 |

*Note.* Anxiety, arousal, disgust: range 1- 7, low values equal low rating, valence: range -3 – +3, *M* = mean, *SD* = standard deviation.

**Table S3**

*Ratings per motion*

|  | anxiety | | arousal | | disgust | | valence | |
| --- | --- | --- | --- | --- | --- | --- | --- | --- |
| motion | *M* | *SD* | *M* | *SD* | *M* | *SD* | *M* | *SD* |
| pictures | 1.84 | 0.68 | 1.92 | 0.75 | 2.09 | 0.69 | +1.08 | 0.89 |
| videos | 1.90 | 0.70 | 2.10 | 0.89 | 2.19 | 0.70 | +1.19 | 0.84 |

*Note.* Anxiety, arousal, disgust: range 1- 7, low values equal low rating, valence: range -3 – +3, *M* = mean, *SD* = standard deviation.

**Table S4**

*Ratings per motion type*

|  | anxiety | | arousal | | disgust | | valence | |
| --- | --- | --- | --- | --- | --- | --- | --- | --- |
| motion type | *M* | *SD* | *M* | *SD* | *M* | *SD* | *M* | *SD* |
| natural | 2.02 | 0.71 | 2.19 | 0.87 | 2.36 | 0.70 | +1.03 | 0.76 |
| point-light | 1.73 | 0.69 | 1.85 | 0.77 | 1.92 | 0.73 | +1.23 | 0.99 |

*Note.* Anxiety, arousal, disgust: range 1- 7, low values equal low rating, valence: range -3 – +3, *M* = mean, *SD* = standard deviation.

**Table S5**

*Anxiety ratings per animal and motion*

|  | pictures | | videos | |
| --- | --- | --- | --- | --- |
| anxiety | *M* | *SD* | *M* | *SD* |
| cat | 1.24 | 0.55 | 1.17 | 0.46 |
| dove | 1.11 | 0.28 | 1.10 | 0.34 |
| snake | 1.77 | 0.90 | 1.83 | 1.00 |
| spider | 3.25 | 1.77 | 3.51 | 1.91 |

*Note.* *M* = mean, *SD* = standard deviation. Anxiety: range 1 - 7.

**Table S6**

*Arousal ratings per animal and motion*

|  | pictures | | videos | |
| --- | --- | --- | --- | --- |
| Arousal | *M* | *SD* | *M* | *SD* |
| cat | 1.41 | 0.68 | 1.40 | 0.86 |
| dove | 1.21 | 0.44 | 1.31 | 0.75 |
| snake | 1.76 | 0.91 | 1.97 | 1.13 |
| spider | 3.30 | 1.82 | 3.75 | 3.75 |

*Note.* *M* = mean, *SD* = standard deviation. Arousal: range 1 - 7.

**Table S7**

*Disgust ratings per animal and motion*

|  | pictures | | videos | |
| --- | --- | --- | --- | --- |
| disgust | *M* | *SD* | *M* | *SD* |
| cat | 1.19 | 0.40 | 1.12 | 0.30 |
| dove | 1.47 | 0.60 | 1.45 | 0.65 |
| snake | 1.74 | 0.97 | 1.90 | 1.13 |
| spider | 3.97 | 1.87 | 4.29 | 1.89 |

*Note.* *M* = mean, *SD* = standard deviation. Disgust: range 1 - 7.

**Table S8**

*Valence ratings per animal and motion*

|  | pictures | | videos | |
| --- | --- | --- | --- | --- |
| valence | *M* | *SD* | *M* | *SD* |
| cat | +2.17 | 0.89 | +2.4 | 0.83 |
| dove | +1.58 | 1.16 | +1.9 | 1.13 |
| snake | +1.21 | 1.20 | +1.29 | 1.26 |
| spider | -0.63 | 1.50 | -0.80 | 1.56 |

*Note.* *M* = mean, *SD* = standard deviation. Valance range -3 – +3.

**Table S9**

*Anxiety ratings per animal and motion type*

|  | natural | | point-light | |
| --- | --- | --- | --- | --- |
| anxiety | *M* | *SD* | *M* | *SD* |
| cat | 1.12 | 0.41 | 1.29 | 0.59 |
| dove | 1.12 | 0.31 | 1.10 | 0.30 |
| snake | 2.04 | 3.79 | 1.56 | 0.83 |
| spider | 3.79 | 1.96 | 2.98 | 1.79 |

*Note.* *M* = mean, *SD* = standard deviation. Anxiety: range 1 - 7.

**Table S10**

*Arousal ratings per animal and motion type*

|  | natural | | point-light | |
| --- | --- | --- | --- | --- |
| arousal | *M* | *SD* | *M* | *SD* |
| cat | 1.42 | .90 | 1.39 | .68 |
| dove | 1.30 | .63 | 1.22 | .50 |
| snake | 2.11 | 1.18 | 1.62 | .91 |
| spider | 3.88 | 1.97 | 3.18 | 1.87 |

*Note.* *M* = mean, *SD* = standard deviation. Arousal: range 1 - 7.

**Table S11**

*Disgust ratings per animal and motion type*

|  | natural | | point-light | |
| --- | --- | --- | --- | --- |
| disgust | *M* | *SD* | *M* | *SD* |
| cat | 1.07 | 0.21 | 1.24 | 0.48 |
| dove | 1.67 | 0.89 | 1.25 | 0.46 |
| snake | 1.99 | 1.16 | 1.65 | 0.96 |
| spider | 4.71 | 1.90 | 3.56 | 1.99 |

*Note.* *M* = mean, *SD* = standard deviation. Disgust: range 1 - 7.

**Table S12**

*Valence ratings per animal and motion type*

|  | natural | | point-light | |
| --- | --- | --- | --- | --- |
| valence | *M* | *SD* | *M* | *SD* |
| cat | +2.55 | 0.68 | +1.98 | 1.08 |
| dove | +1.62 | 1.16 | +1.87 | 1.21 |
| snake | +1.10 | 1.28 | +1.40 | 1.21 |
| spider | -1.14 | 1.50 | -0.30 | 1.61 |

*Note.* *M* = mean, *SD* = standard deviation. Valance range -3 – +3.

**Table S13**

*Anxiety ratings per animal and group*

|  | non-spider-fearful | | spider-fearful | |  |
| --- | --- | --- | --- | --- | --- |
| anxiety | *M* | *SD* | *M* | *SD* | *p* |
| cat | 1.25 | 0.61 | 1.14 | 0.23 | .366 |
| dove | 1.13 | 0.39 | 1.08 | 0.13 | .587 |
| snake | 1.84 | 0.96 | 1.77 | 0.91 | .792 |
| spider | 2.10 | 1.13 | 4.74 | 1.35 | < .001 |

*Note.* *p* = p-value of four-way mixed ANOVA. *M* = mean, *SD* = standard deviation. Anxiety: range 1 - 7.

**Table S14**

*Arousal ratings per animal and group*

|  | non-spider-fearful | | spider-fearful | |  |
| --- | --- | --- | --- | --- | --- |
| arousal | *M* | *SD* | *M* | *SD* | *p* |
| cat | 1.50 | 0.87 | 1.32 | 0.60 | .357 |
| dove | 1.32 | 0.70 | 1.32 | 0.34 | .437 |
| snake | 1.90 | 1.09 | 1.82 | 0.89 | .822 |
| spider | 2.19 | 1.22 | 4.91 | 1.39 | < .001 |

*Note.* *p* = p-value of four-way mixed ANOVA. *M* = mean, *SD* = standard deviation. Arousal: range 1-7.

**Table S15**

*Disgust ratings per animal and group*

|  | non-spider-fearful | | spider-fearful | |  |
| --- | --- | --- | --- | --- | --- |
| disgust | *M* | *SD* | *M* | *SD* | *p* |
| cat | 1.14 | 0.34 | 1.16 | 0.25 | .793 |
| dove | 1.47 | 0.64 | 1.44 | 0.54 | .854 |
| snake | 1.89 | 1.01 | 1.75 | 1.05 | .590 |
| spider | 2.77 | 1.33 | 5.54 | 1.13 | < .001 |

*Note.* *p* = p-value of four-way mixed ANOVA. *M* = mean, *SD* = standard deviation. Disgust: range 1-7.

**Table S16**

*Valence ratings per animal and group*

|  | non-spider-fearful | | spider-fearful | |  |
| --- | --- | --- | --- | --- | --- |
| valence | *M* | *SD* | *M* | *SD* | *p* |
| cat | + 2.07 | 0.91 | + 2.46 | 0.61 | .050 |
| dove | + 1.43 | 1.14 | + 2.07 | 0.97 | .022 |
| snake | + 0.86 | 1.12 | + 1.65 | 1.16 | .007 |
| spider | + 0.22 | 1.25 | - 1,7 | 1.05 | < .001 |

*Note.* *p* = p-value of four-way mixed ANOVA. *M* = mean, *SD* = standard deviation. Valance range -3 – +3.

**Table S17**

*Anxiety ratings per animal, motion type and motion*

| anxiety | natural | | point-light | |
| --- | --- | --- | --- | --- |
|  | *M* | *SD* | *M* | *SD* |
| pictures | | | | |
| cat | 1.11 | 0.41 | 1.37 | 0.82 |
| dove | 1.13 | 0.37 | 1.09 | 0.25 |
| snake | 2.01 | 1.10 | 1.53 | 0.86 |
| spider | 3.71 | 1.91 | 2.79 | 1.78 |
| videos | | | | |
| cat | 1.13 | 0.41 | 1.20 | 0.54 |
| dove | 1.10 | 0.30 | 1.11 | 0.40 |
| snake | 2.08 | 1.13 | 1.59 | 0.97 |
| spider | 3.86 | 2.06 | 3.16 | 1.90 |

*Note.* *M* = mean, *SD* = standard deviation. Anxiety: range 1 - 7.

**Table S18**

*Arousal ratings per animal, motion type and motion*

| arousal | natural | | point-light | |
| --- | --- | --- | --- | --- |
|  | *M* | *SD* | *M* | *SD* |
| Pictures | | | | |
| cat | 1.39 | .81 | 1.43 | .73 |
| dove | 1.26 | .58 | 1.15 | .36 |
| snake | 1.96 | 1.08 | 1.56 | .89 |
| spider | 3.73 | 1.93 | 2.88 | 1.80 |
| videos | | | | |
| cat | 1.45 | 1.02 | 1.36 | .76 |
| dove | 1.34 | .80 | 1.28 | .73 |
| snake | 2.26 | 1.34 | 1.68 | 1.08 |
| spider | 4.02 | 2.05 | 3.48 | 2.03 |

*Note.* *M* = mean, *SD* = standard deviation. Arousal: range 1 - 7.

**Table S19**

*Disgust ratings per animal, motion type and motion*

| disgust | natural | | point-light | |
| --- | --- | --- | --- | --- |
|  | *M* | *SD* | *M* | *SD* |
| pictures | | | | |
| cat | 1.33 | 0.80 | 1.72 | 0.91 |
| dove | 1.2 | 0.48 | 1.91 | 1.13 |
| snake | 1.57 | 0.94 | 4.62 | 1.93 |
| spider | 4.62 | 1.93 | 3.33 | 2.02 |
| videos | | | | |
| cat | 1.08 | 0.28 | 1.15 | 0.41 |
| dove | 1.63 | 0.93 | 1.27 | 0.64 |
| snake | 2.07 | 1.23 | 1.73 | 1.15 |
| spider | 4.80 | 1.92 | 3.78 | 2.10 |

*Note.* *M* = mean, *SD* = standard deviation. Disgust: range 1 - 7.

**Table S20**

*Valence ratings per animal, motion type and motion*

| valence | natural | | point-light | |
| --- | --- | --- | --- | --- |
|  | *M* | *SD* | *M* | *SD* |
| pictures | | | | |
| cat | +2.50 | .76 | +1.85 | 1.26 |
| dove | +1.46 | 1.21 | +1.77 | 1.30 |
| snake | +1.10 | 1.28 | +1.32 | 1.32 |
| spider | -1.09 | 1.49 | -0.17 | 1.65 |
| videos | | | | |
| cat | +2.62 | .65 | +2.10 | 1.16 |
| dove | +1.76 | 1.18 | +2.04 | 1.25 |
| snake | +1.09 | 1.32 | +1.50 | 1.37 |
| spider | -0.18 | 1.55 | -0.43 | 1.73 |

*Note.* *M* = mean, *SD* = standard deviation. Disgust: range 1 - 7. Valance range -3 – +3.

**Table S21**

*Reaction times per target/distractor types*

| target/distractors type | *M* | *SD* |
| --- | --- | --- |
| snake target/neutral distractors | 0.896 | 0.261 |
| neutral target/snake distractors | 1.080 | 0.227 |
| neutral target/neutral distractors | 1.110 | 0.256 |
| neutral target/spider distractors | 1.190 | 0.280 |
| spider target/neutral distractors | 1.120 | 0.264 |

*Note.* Reaction times in seconds. *M* = mean, *SD* = standard deviation.

**Table S22**

*Reaction times per target/distractor types and group*

|  | spider-fearful | | non-spider-fearful | |
| --- | --- | --- | --- | --- |
| target/distractors type | *M* | *SD* | *M* | *SD* |
| snake target/  neutral distractors | 0.893 | 0.263 | 0.899 | 0.258 |
| neutral target/  snake distractors | 1.100 | 0.243 | 1.060 | 0.208 |
| neutral target/  neutral distractors | 1.110 | 0.253 | 1.110 | 0.260 |
| neutral target/  spider distractors | 1.210 | 0.303 | 1.160 | 0.255 |
| spider target/  neutral distractors | 1.120 | 0.279 | 1.130 | 0.248 |

*Note.* Reaction times in seconds. *M* = mean, *SD* = standard deviation.

**Table S23**

*Reaction times per motion*

| Motion | *M* | *SD* |
| --- | --- | --- |
| target static/ distractors move | 1.170 | 0.295 |
| target static/ distractors static | 1.090 | 0.278 |
| target moves/ distractors static | 0.973 | 0.210 |

*Note.* Reaction times in seconds. *M* = mean, *SD* = standard deviation.

**Table S24**

*Reaction times per target/distractor types and motion*

| target/distractors type | target static/ distractors move | | target static/ distractors static | | target moves/ distractors static | |
| --- | --- | --- | --- | --- | --- | --- |
|  | *M* | *SD* | *M* | *SD* | *M* | *SD* |
| snake target/  neutral distractors | 0.969 | 0.283 | 0.868 | 0.231 | 0.852 | 0.253 |
| neutral target/  snake distractors | 1.120 | 0.255 | 1.130 | 0.214 | 0.986 | 0.176 |
| neutral target/  neutral distractors | 1.250 | 0.265 | 1.070 | 0.244 | 1.020 | 0.198 |
| neutral target/  spider distractors | 1.310 | 0.275 | 1.230 | 0.292 | 1.020 | 0.170 |
| spider target/  neutral distractors | 1.230 | 0.270 | 1.140 | 0.263 | 0.996 | 0.198 |

*Note.* Reaction times in seconds. *M* = mean, *SD* = standard deviation.

**Table S25**

*Reaction times per motion type*

| motion type | *M* | *SD* |
| --- | --- | --- |
| natural | 1.200 | 0.249 |
| point-light | 0.959 | 0.248 |

*Note.* Reaction times in seconds. *M* = mean, *SD* = standard deviation.

**Table S26**

*Reaction times per target/distractor types, motion and motion type*

| target/distractors type | target static/ distractors move | | target static/ distractors static | | target moves/ distractors static | |
| --- | --- | --- | --- | --- | --- | --- |
|  | *M* | *SD* | *M* | *SD* | *M* | *SD* |
| Natural | | | | | | |
| snake target/  neutral distractors | 1.150 | 0.249 | 1.020 | 0.203 | 1.040 | 0.210 |
| neutral target/  snake distractors | 1.240 | 0.231 | 1.190 | 0.218 | 1.080 | 0.169 |
| neutral target/  neutral distractors | 1.390 | 0.236 | 1.220 | 0.197 | 1.130 | 0.178 |
| neutral target/  spider distractors | 1.440 | 0.234 | 1.381 | 0.258 | 1.110 | 0.155 |
| spider target/  neutral distractors | 1.274 | 0.253 | 1.222 | 0.262 | 1.108 | 0.175 |
| point-light | | | | | | |
| snake target/  neutral distractors | 0.789 | 0.181 | 0.715 | 0.138 | 0.666 | 0.121 |
| neutral target/  snake distractors | 0.994 | 0.217 | 1.073 | 0.195 | 0.896 | 0.132 |
| neutral target/  neutral distractors | 1.108 | 0.215 | 0.917 | 0.189 | 0.902 | 0.144 |
| neutral target/  spider distractors | 1.177 | 0.250 | 1.088 | 0.248 | 0.922 | 0.128 |
| spider target/  neutral distractors | 1.192 | 0.281 | 1.065 | 0.242 | 0.885 | 0.152 |

*Note.* Reaction times in seconds. *M* = mean, *SD* = standard deviation.

**C: Exploratory analyses**

***Stimulus ratings do not affect target reaction times***

When correlating the individual mean ratings (anxiety, arousal, valence, disgust) for spiders and snakes with the mean reaction time for spider and snake targets, we did not obtain any significant correlation (all *p* > .09, see Fig. S1). However, we observed a small, but non-significant positive correlation between disgust as well as valence ratings and the reaction time to snake targets. Future studies with larger sample sizes might aim to replicate and validate this relationship. Even if the correlation does not reach significance, it might point toward a positive relationship between disgust and valence and attentional capture of snakes, i.e. the more positive and less disgusting snakes are perceived, the faster the reaction times. Such a relationship was not present among spider targets.

Furthermore, Fig. S1 shows, that we did only recruit individuals with snake fear below or equal to the 50^th^ percentile as intended, since ratings rarely exceeded medium values (4).


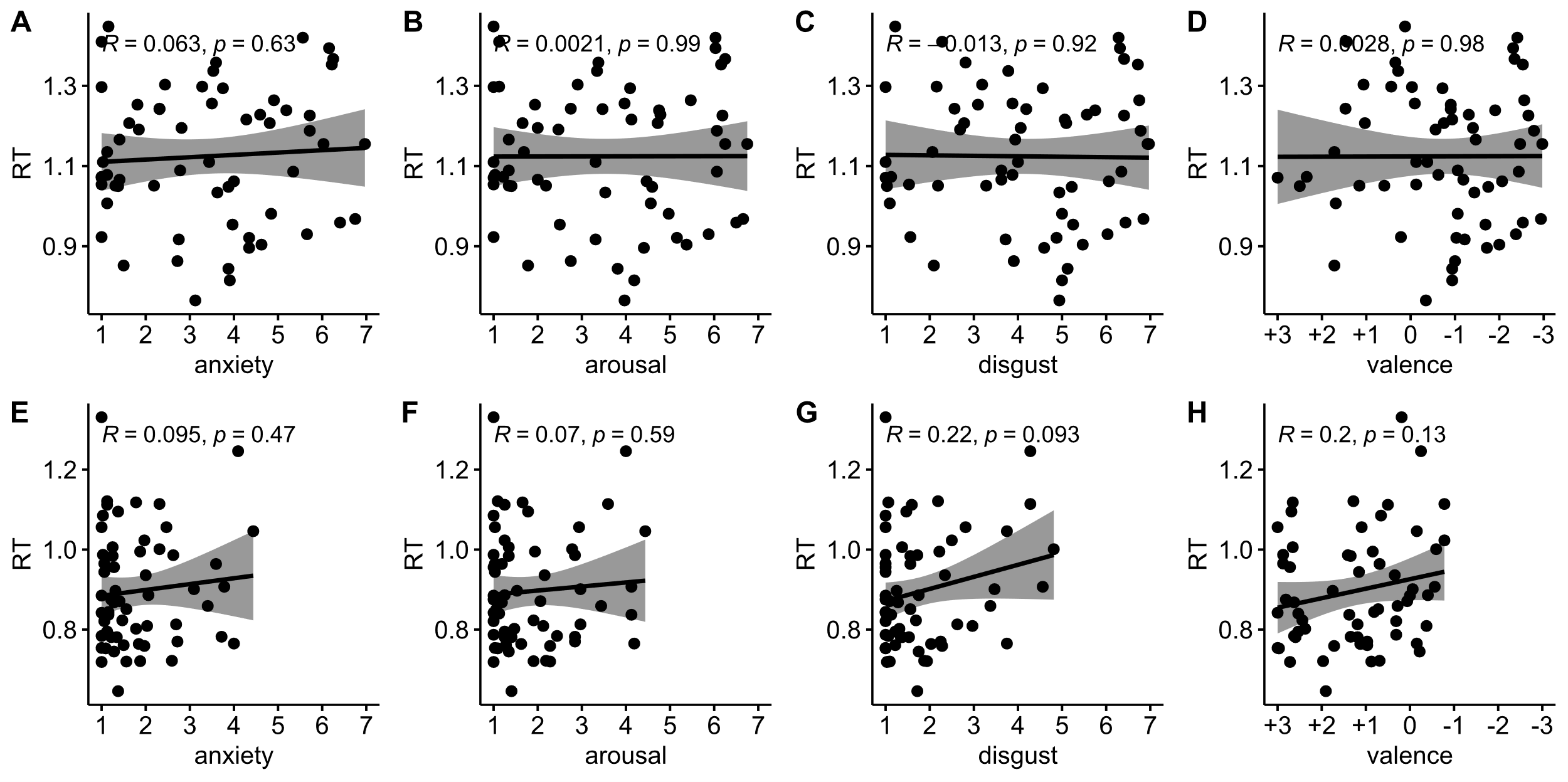
**Figure S1.** Scatter plots of mean ratings of snake/spider stimuli and reaction times per participant. A-D show scatterplots for spider target stimuli. Plot E-H shows scatterplots for snake target stimuli. *R* = pearson correlation, *p* = p-value.

***Target/distractor type effects across motion conditions***

**(1a) Snake but not spider targets speed up search irrespective of spider-fearfulness.** Target/distractor valence significantly affected reaction times (*F*_(3.16, 186.22)_ = 126.98, *p* < .001). Comparable to the results in the static condition, snake targets but not spider targets elicited faster reaction times than neutral targets across all motion conditions (snake target: *t*_(365)_ = -18.7, *p* < .001; spider target: *t*_(365)_ = -1.2, *p* = .232), contradicting our assumption that spider targets, too, would speed up search across groups (see Figure S2).

The group × target/distractors type interaction was almost significant (*F*_(3.16 186.22 )_ = 2.52, *p* = .056), but no main effect of group was found within any of the different target/distractors types (*p* > .1). This implies that spider-fearfulness did not modulate the reactions to different target/distractor combinations. However, within each group the main effect of target/distractors type was significant (*F*_(2.83, 85)_ = 62.6, *p* < .001, F_(2.99, 86.7)_ = 66.3, *p* < .001, Figure S2). Specifically, both groups identified snake targets faster than neutral targets (*t*_(185)_ = -12.6, *t*_(180)_ = -14, *p* < .001) – but in contrast to our hypothesis, spider targets were not found faster than neutral targets, irrespective of group (*t*_(185)_ = -1.27, *t*_(180)_ = -0.444, *p* > .01). In fact, spider-fearful participants were even slower in finding spider compared to snake targets (*t*_(180)_ = -10.8, *p* < .001).


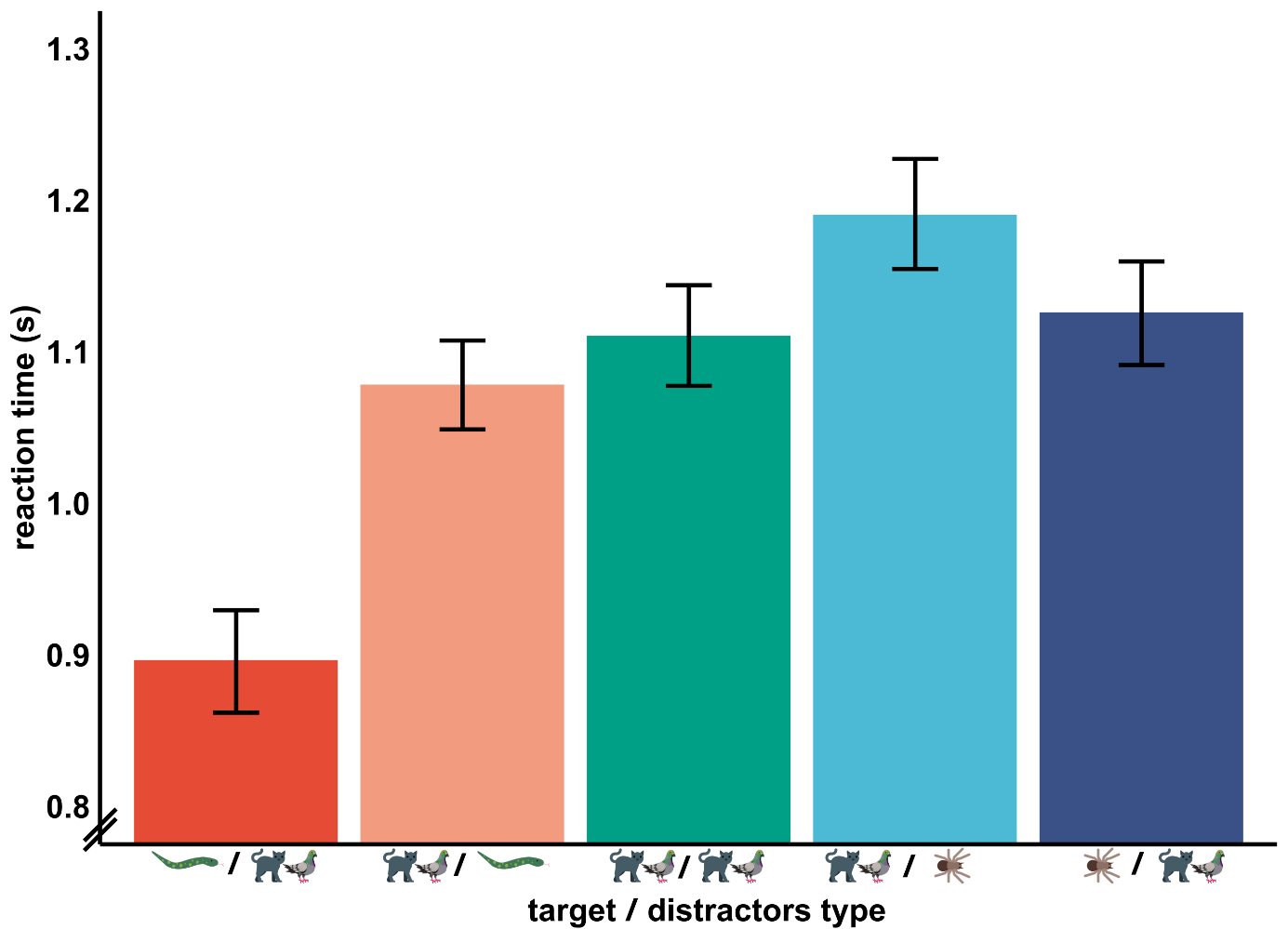


**Figure S2.** *Target/distractors type differences across groups and all motion conditions. Note.* Mean ± SEM.

**(1b) Spider distractors slow down and snake distractors speed up search irrespective of spider-fearfulness.** As expected, spider distractors slowed down search compared to neutral distractors (*t*_(365)_ = -6.91, *p* < .001), showing an increased distraction by spiders (see Figure S2). Unexpectedly, snake distractors sped up search compared to neutral distractors (*t*_(365)_ = -2.88, *p* =.004). In line with our hypotheses, spider distractors slowed down search in both groups (non-fearful group: *t*_(185)_ = -3.33, *p* = .001; spider-fearful group: *t*_(179)_ = -6.4, *p* < .001). Importantly, however, spider-fearful individuals were not more strongly distracted than non-fearful-individuals (*F*_(1, 59)_ = 1.61, *p* = .209). At the same time, non-spider-fearful participants reacted not slower but faster to target/distractors types with snake distractors (*t*_(185)_ = -3.59, *p* < .001), which was not the case for spider-fearful participants (*t*_(179)_ = -0.566, *p* = .602).

***Motion type affects the motion and target/distractors type interaction***

Motion type did not influence the effect of motion. We also did not detect an effect of motion on the interaction of group × target/distractors type (*p* > .05). The three-way interaction motion type × motion × target/distractors type was significant (*p* < .001), illustrating that presenting point-light stimuli vs. natural videos had an effect on the motion × target/distractors type effect First, we will describe the influence of motion type on the simple main effects of motion within different target/distractors types.

The absence of an additional motion effect on the identification of snake targets (*t*_(60)_ = -0.916, *p* = .363). and targets among snake distractors (*t*_(60)_ = 1.90, *p* = .069) was present in natural stimuli, but, point-light stimuli movement enhanced the effects (*t*_(60)_ = 3.57, *p* < .001, *t*_(60)_ = -3.17, *p* = .003). If the point-light display consisted of only neutral stimuli, target movement did not speed up the search compared to static motion types (*t*_(60)_ = 0.965, *p* = .351). Within the natural stimuli, presenting moving compared to static spider stimuli (either as target or distractor) did influence reaction times (*t*_(60)_ = 4.85, *p* < .001, *t*_(60)_ = 2.24, *p* = .034). The same was true for point-light stimuli (*t*_(60)_ = 7.55, *p* < .001, *t*_(60)_ = 3.51, *p* = .001). In summary, when isolating “pure” animal motion by using point-light stimuli, motion had an additional effect on the reaction times to snake and spiders (as targets or distractors).

Second, we will focus on the influence of motion type on the simple main effects of target/distractors types within different motion conditions. In static motion condition, snake targets sped up search (natural: *t*_(60)_ = -6.85, *p* < .001, point-light: *t*_(60)_ = -8.47, *p* < .001)., while spider distractors slowed down search for both motion types. (natural: *t*_(60)_ = -5.55, *p* < .001, point-light: *t*_(60)_ = -5.82, *p* < .001). Static spider targets on the other hand only slowed down search, when they were point-light (*t*_(60)_ = -5.29, *p* < .001) and not pictures (*t*_(60)_ = -0.185, *p* = .868).

Finally, static snake distractors did not speed up search compared to neutral target/distractor types (*t*_(60)_ = -0.862, *p* = .454), if they were natural. Thus, the previously reported speeded detection of targets among snake distractors does only occur with static point-light stimuli. In a nutshell, snake targets were detected faster and spider distractors slowed down search independent of motion or motion type. In contrast, distraction by static snakes and the slowdown of spider targets only appear among point-light stimuli, meaning the results from previous studies are likely attributed to the simplicity of stimuli (no background, little detail, or color).

For both motion conditions, the reversed effect of snake distractors on reaction time for moving distractors was found (*t*_(60)_ = -4.95, *p* < .001, *t*_(60)_ = -4.8, *p* < .001), i.e. moving snake distractors speed up search. Therefore, this reversal is likely due to biological motion. The distraction by spiders was only significant among point-light stimuli (*t*_(60)_ = -3.16, *p* = .009), but not natural stimuli (*t*_(60)_ = -1.72 *p* = .131) This is possibly due to increased reaction times to natural target/distractors types, as visible in Figure 2.

Effects among moving target/static distractors types were similar for both natural and point light motion type. Once targets moved, all differences between different target/distractors types disappeared except for the speeded detection of snake targets (presented as point-light stimuli or natural videos: *t*_(60)_ = -13.0, *p* < .001; *t*_(60)_ = -3.76, *p* < .001). Among natural motion target/distractors types snake distractors sped up search (*t*_(60)_ = -2.4, *p* = .03), meaning here the effect was not overridden by motion. Among point-light stimuli, spider distractors slowed down search more than spider targets, if targets moved (*t*_(60)_ = 2.23, *p* = .044).

## References

1. Cohen, J. *Statistical Power Analysis for the Behavioural Sciences.* (Laurence Erlbaum Associates. Inc., 1988).

2. Burra, N., Pittet, C., Barras, C. & Kerzel, D. Attentional suppression is delayed for threatening distractors. *Vis. Cogn.* **27**, 185–198 (2019).

3. Miltner, W. H. R., Krieschel, S., Hecht, H., Trippe, R. & Weiss, T. Eye movements and behavioral responses to threatening and nonthreatening stimuli during visual search in phobic and nonphobic subjects. *Emotion* **4**, 323–339 (2004).

4. Rinck, M., Reinecke, A., Ellwart, T., Heuer, K. & Becker, E. S. Speeded Detection and Increased Distraction in Fear of Spiders: Evidence From Eye Movements. *J. Abnorm. Psychol.* **114**, 235–248 (2005).

5. Caudek, C., Ceccarini, F. & Sica, C. Facial expression movement enhances the measurement of temporal dynamics of attentional bias in the dot-probe task. *Behav. Res. Ther.* **95**, 58–70 (2017).
